# The Precision and Safety of Ultrasound-Guided versus Palpation-Guided Needle Placement on the Plantar Fascia and Flexor Digitorum Brevis Interface: An Anatomical Study

**DOI:** 10.3390/healthcare12101000

**Published:** 2024-05-13

**Authors:** Miguel Malo-Urriés, Sergio Borrella-Andrés, Carlos López-de-Celis, César Fernández-de-las-Peñas, Albert Pérez-Bellmunt, José L. Arias-Buría, Isabel Albarova-Corral, Jacobo Rodríguez-Sanz

**Affiliations:** 1 Health Sciences Faculty, Department of Physiatry and Nursing, University of Zaragoza, 50009 Zaragoza, Spain; malom@unizar.es (M.M.-U.); sergiocai04@gmail.com (S.B.-A.); ialbarova@unizar.es (I.A.-C.); 2 Faculty of Medicine and Health Sciences, Universitat International de Catalunya, 08195 Sant Cugat del Vallès, Spain; carlesldc@uic.es (C.L.-d.-C.); aperez@uic.cat (A.P.-B.); jrodriguezs@uic.es (J.R.-S.); 3 ACTIUM Functional Anatomy Group, 08195 Sant Cugat del Vallès, Spain; 4 Fundació Institut, Universitari per a La Recerca a l’Atenció, Primària de Salut Jordi Gol i Gurina (IDIAPJGol), 08028 Barcelona, Spain; 5 Department of Physical Therapy, Occupational Therapy, Rehabilitation and Physical Medicine, Universidad Rey Juan Carlos, 28922 Alcorcón, Spain; joseluis.arias@urjc.es

**Keywords:** plantar fascia, ultrasound, needling, interphase, accuracy, safety, cadaver

## Abstract

Background: Evidence suggests the plantar fascia and its interphase with the flexor digitorum brevis muscle can play a relevant role in plantar heel pain. Needling interventions could offer an appropriate treatment strategy to addressing this interface. Objective: We compared the accuracy and safety of ultrasound-guided versus palpation-guided procedures for the proper targeting of the interface between the plantar fascia and the flexor digitorum brevis with a solid needle. Methods: A crossover cadaveric study was conducted. Five experienced therapists performed a series of 20 needle insertions each (n = 100 in total, 10 landmark-guided and 10 ultrasound-guided) on 10 anatomical samples. The therapists were instructed to accurately place the needle on the interface between the plantar fascia and the flexor digitorum brevis muscle. The distance of the tip of the needle to the identified target (accuracy), the surrounding sensitive structures targeted (safety), the time needed for the procedure, the number of needle passes, and the needle length outside the skin were assessed. Results: The ultrasound-guided technique was associated with a significantly higher accuracy (*p* < 0.001) but without differences in safety (*p* = 0.249) as compared to the palpation-guided procedure. Conclusion: Our results suggest that ultrasound-guided insertion exhibits greater accuracy but not greater safety than palpation-guided insertion when targeting the interface between the plantar fascia and the flexor digitorum brevis.

## 1. Introduction

Plantar heel pain or plantar fasciitis is one of the most prevalent tendinopathies of the lower extremity, as it affects 20–40% of both athletic [1] and non-athletic [2] populations. Conservative management is considered the first therapeutic line for treating plantar heel pain. Clinical guidelines recommend different treatment interventions, e.g., corticosteroid injection, exercises, extracorporeal shockwave therapy, or manual therapies for plantar heel pain [3,4]; however, current evidence on which treatment strategy is the most effective is inconclusive [5]. Two meta-analyses have found that needling interventions are effective for managing plantar heel-associated pain [6] but highlighted the need for further research on their efficacy and the methodology used to perform these interventions.

It has recently been suggested that a relationship exists between plantar fascia involvement and the flexor digitorum brevis [7]. It was proposed that involvement of the flexor digitorum brevis muscle may be a factor that increases the likelihood of experiencing plantar heel pain [7]. This hypothesis would support the use of interventions targeting the interphase between these two structures. In fact, the role of soft-tissue interphases in other body areas, such as the Hoffa’s fat pad and the patellar tendon for knee pain problems, has been investigated [8].

Percutaneous needle electrolysis is a technique that involves the application of a galvanic electric current delivered through a filiform needle and is commonly used in the clinic to treat connective tissue at these interfaces [9]. This intervention generates a controlled inflammatory response in a specific target tissue without an increase in temperature [10], allowing phagocytosis of the degenerated tissue and subsequent targeted repair [9]. This intervention has been shown to be safe and non-thermal without provoking a loss of metal particles or modifying the morphology of the needles used when studied in vitro [11].

Moderate quality evidence supports a positive effect of percutaneous needle electrolysis for reducing pain and related-disability in chronic pain conditions of musculoskeletal origin [12]. In fact, percutaneous needle electrolysis can be applied to different tissues such as tendons [13], muscles [14], or nerves [15]. Further, this intervention has been advocated for managing scars or connective tissue at different interphases, e.g., hamstring tendon–sciatic nerve [14]. Accordingly, accurate and safe needle procedures targeting specific tissue-to-tissue interphases are needed.

Needling interventions can be performed based on anatomical landmarks (palpation-guided procedure) or by using an ultrasound equipment (ultrasound-guided procedure). In fact, needling interventions traditionally depend on manual palpation and anatomical landmarks, and their accuracy is related to the therapist skill level. Nevertheless, the use of imaging procedures has led to the adoption of guidance methods aimed at enhancing precision and mitigating adverse effects associated with needling interventions. The American Society of Regional Anesthesia and Pain Medicine (ASRA) considers inaccurate needle positioning as a major cause of the limited clinical effectiveness of invasive procedures and potential adverse events [16]. Ultrasound offers real-time guidance, thereby facilitating accurate needle placement, and is able to decrease the likelihood of inadvertently puncturing sensitive tissues [17,18,19]. However, no study has compared the accuracy and safety of ultrasound-guided vs. palpation-guided procedures for needle placement at the interface between the plantar fascia and the flexor digitorum brevis. Therefore, the aims of the current study were (1) to compare ultrasound-guided against palpation-guided needling procedure in terms of accuracy, safety, and performance when targeting the interface between the plantar fascia and the flexor digitorum brevis and (2) to assess the differences between using and not using the handpiece, the same one used during the application of percutaneous needle electrolysis, to perform the procedure on a cadaveric model.

## 2. Methods

### 2.1. Study Design

A cross-sectional anatomical study on 10 cryopreserved specimens was conducted. The study obtained the Local Ethics Committee approval from the Universitat Internacional de Catalunya (CBAS-2021-09). Five physical therapists with more than 10 years of experience in needling interventions performed a total of 20 needle insertions each (n = 100), 10 palpation-guided (n = 50) and 10 ultrasound-guided (n = 50).

Ten frozen anatomical samples were stored under refrigerated conditions (−20 °C) and thawed to ambient temperature for at least 24 h before the procedure to maintain normal tissue characteristics. All participants underwent a 10 min standardized instructional and practical session before the protocol commenced to understand the study’s purpose and become familiar with the procedure of the study [20].

### 2.2. Procedure

Therapists were instructed to place the body of the needle in the interface between the plantar fascia and flexor digitorum brevis of the cadaveric model, at the nearest point of the plantar fascia at the insertion on the calcaneus by applying as many needles passes as necessary until they considered the needle placement satisfactory [19].

The needling was conducted via both palpation-guided (anatomical landmarks) and ultrasound-guided procedures. Each therapist completed a total of 20 needle insertions (10 palpation-guided and 10 ultrasound-guided) with a short wash-out break period after each procedure and a 5 min break rest after 10 attempts to prevent fatigue [20,21]. The palpation-guided approach was first conducted before the ultrasound-guided approach for avoiding pre-visualization with the ultrasound that could assist with a posterior palpation-guided approach.

#### 2.2.1. The Palpation-Guided Approach

**Figure 1:**
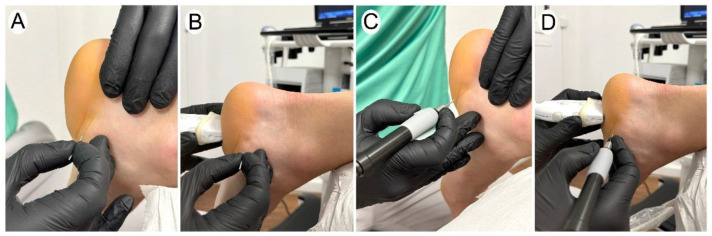
Application of the needling procedure on the interface formed between the plantar fascia and the flexor digitorum brevis muscle. (**A**) Palpation-guided needle procedure without a handpiece, (**B**) ultrasound-guided needle procedure without a handpiece, (**C**) palpation-guided needling procedure with a handpiece, and (**D**) ultrasound-guided needling procedure with a handpiece.

**Figure 2:**
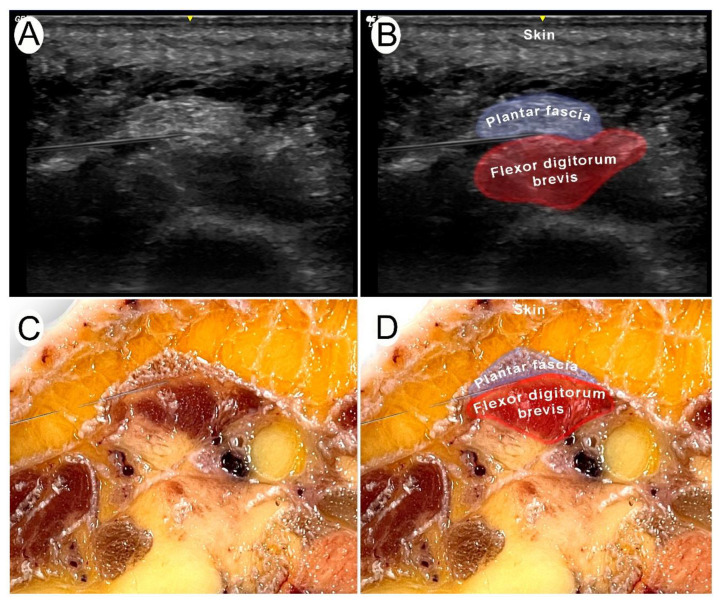
Needling intervention of the interface formed between the plantar fascia and the flexor digitorum brevis muscle with surrounding structures. (**A**) Ultrasound image for the needling procedure, (**B**) ultrasound identification of the structures for measurements, (**C**) cross-sectional image of the interface formed between the plantar fascia and the flexor digitorum brevis muscle in a cadaver with the needle reaching the targeted zone, and (**D**) cadaveric identification of the plantar fascia and the flexor digitorum brevis muscle with the tip of the needle in the targeted point.

Participants were asked to complete the task with the sole guidance of their palpatory skills. The anterior and inferior limits of the calcaneus bone were identified. The needle was introduced with the dominant hand from the medial side into the proximal portion of the plantar fascia, at the closest point of the plantar fascia to the insertion in the calcaneal bone (Figure 1A). The target was to place the needle into the interface formed between the plantar fascia and the flexor digitorum brevis (Figure 2A).

#### 2.2.2. The Ultrasound-Guided Approach

A LOGIQ eR8 (General Electric Healthcare) ultrasound scanner with a 4–12 MHz linear transducer was used. An ultrasonographic image was pre-calibrated and optimized for basic parameters (frequency, depth, gain, and focus) by an external researcher in a standardized manner to allow therapists to focus on the approach. Therapists were instructed to perform the ultrasound-guided technique in an “in-plane” approach, with a cross-sectional view of the plantar fascia and flexor digitorum brevis (Figure 2A). The probe, held in the non-dominant hand, was placed in the sagittal plane of the heel to identify from superficial to deep, the infracalcaneal fat pad, plantar fascia, calcaneus and flexor digitorum brevis (Figure 1B). Once optimal visualization of these structures was obtained, the needle was introduced with the dominant hand from the medial side. The objective was to introduce the needle between the plantar fascia and the flexor digitorum brevis (Figure 2B).

Both conditions were performed with a filiform solid needle (5 palpation-guided attempts, as shown in Figure 1A, and 5 ultrasound-guided attempts, as shown in Figure 1B) and with a filiform needle inserted into a handpiece (5 palpation-guided attempts, as shown in Figure 1C, and 5 ultrasound-guided attempts, as shown in Figure 1D). The choice of using the handheld was randomly selected by using a computerized random assignment list. All tasks were conducted under the same condition, with a fixed needle size of 0.30 mm × 40 mm.

### 2.3. Measurements

Accuracy and safety data were considered. Following each needle placement, a researcher with more than 10 years of experience in ultrasound assessment collected the following measurements that were extracted from the ultrasound image: (1) the distance to the interface between the plantar fascia and flexor digitorum brevis (mm) (accuracy), (2) the longitudinal contact of the body of the needle with the interface (mm) (accuracy), (3) the time needed for the procedure (seconds) (accuracy), (4) the number of needle passes (each time a participant advanced the needle after a change of direction was considered one pass) (safety), (5) other structures targeted during the needling insertion, e.g., the plantar fascia or flexor digitorum brevis muscle (safety), and (6) the length of the needle outside the body (mm) (accuracy).

### 2.4. Statistical Analysis

Data were analyzed with IBM SPSS statistics 22.0 software. Descriptive data were expressed as the total number, percentage, mean, and standard deviation (SD). The normal distribution of the variables was analyzed using the Kolmogorov–Smirnov test. Comparative analyses of the quantitative measurements of the palpation-guided and ultrasound-guided procedures and of the procedures with and without the use of the handpiece were performed using independent student t-tests in the case of a normal distribution. In the case of a non-normal distribution, the Mann–Whitney U test was used. The chi-square (χ^2^) test was used to assess the differences in nominal variables. The significance level was set at 0.05. Effect sizes were calculated using Cohen’s d coefficient for quantitative variables. An effect size of >0.8 was considered large, around 0.5 was considered intermediate, and <0.2 was considered small. For qualitative variables, Cramer’s V was used to calculate the effect size. An effect size of >0.5 was considered strong, 0.5–0.3 was considered intermediate, and <0.3 was considered small.

## 3. Results

Clinical characteristics of the five therapists that participated are provided in Table 1. A comparison between landmark-guided and ultrasound-guided procedures is shown in Table 2. The distance from the needle tip to the interface between the plantar fascia and flexor digitorum brevis muscle was significantly lower (*p* < 0.001) with the ultrasound-guided approach (mean: 0.2 ± 0.7 mm) than with the landmark-guided approach (mean: 3.5 ± 2.2 mm). Further, the longitudinal contact of the needle with the interface was also significantly higher (*p* < 0.001) with the ultrasound-guided approach (mean: 5.3 ± 2.2 mm) than with the landmark-guided approach (mean: 0.6 ± 1.8 mm). However, the landmark-guided approach needed a significantly shorter time (mean: 19.1 ± 6.5 vs 53.8 ± 18.9 s, *p* < 0.001), fewer passes (mean: 1.7 ± 0.9 vs 2.8 ± 1.5 in total, *p* < 0.001), and a lower needle length out of the skin (mean: 13.1 ± 3.1) compared to the ultrasound-guided approach (mean: 16.3 ± 2.7, all, *p* < 0.001, Table 2). No significant differences (*p* = 0.249) in those unwanted structures penetrated by a needle during the procedure were identified (Table 2).

Table 3 illustrates the measurements with and without the use of the handpiece with the landmark-guided approach. The use of the handpiece resulted in more time (mean: 21.0 ± 3.7 s) but a greater length of the needle outside the skin (mean: 14.1 ± 2.75 mm) as compared to not using it (mean: 17.1 ± 8.0 s, *p* = 0.029; mean: 16.0 ± 3.2 mm, *p* = 0.01, respectively, Table 3).

Table 4 shows the differences between using or not using the handpiece during the ultrasound-guided approach. It was observed that the use of the handpiece required more time (mean: 59.6 ± 18.4 s) than not using it (mean: 48.1 ± 17.8, *p* = 0.029, Table 4).

## 4. Discussion

This study compared ultrasound-guided versus landmark-guided needling procedure for reaching the interface between the plantar fascia and the flexor digitorum brevis muscle in terms of accuracy and safety and assessed the differences between using and not using a handpiece to perform the approaches on a cadaveric model. The results showed that the ultrasound-guided procedure significantly increased the targeted contact of the needle on the interface compared to the landmark-guided procedure, but a similar rate of success (90% vs. 82%) and safety was achieved.

Previous studies using ultrasound-guidance approaches had attained accuracies ranging from 1.5 to 3.27 mm with different phantoms [17,22,23]. Thus, previous studies performed with a similar methodology but in other body areas such the knee [13] or the elbow [24] also yielded a higher accuracy with ultrasound-guided approaches. In fact, Arias-Buría et al. [13] also evaluated the accuracy of inserting a needle into the interface between the patellar tendon and Hoffa’s fat pad and obtained similar results as in the current study. Arias-Buría et al. [13] also found greater contact of the needle (mean: 15.5 ± 6.65 mm) on the targeted interphase than with the landmark-guided approach (mean: 0.25 ± 0.6 mm). Nevertheless, to achieve this accuracy, the ultrasound-guided approach required significantly more time (mean: 53.8 ± 18.9 s) than the landmark-guided approach (mean: 19.1 ± 6.5 s). Again, these results are also similar to those in previous studies conducted on the knee [13] and elbow [24] regions. Differences in outcomes might occur since ultrasound guidance offers visual cues regarding needle placement, allowing therapists to refine and enhance their technique until reaching the exact target point, thus potentially prolonging the necessary time of the approach. Moreover, it has been demonstrated that using a handpiece also requires additional time and more passes to properly reach the targeted point [13,24].

The rapid advancement of imaging techniques for guiding needle placement, such as ultrasonography, has significantly improved the safety of procedures. Consequently, this enhancement could contribute to heightened patient satisfaction, a decreased prevalence of unintended punctures, and subsequently a broader acceptance and utilization of the technique. In the current study, no differences in terms of safety were found between performing the intervention with or without ultrasound guiding. It is possible that these results were obtained because the adjacent tissues surrounding the plantar fascia are different from the targeted point; hence, through the needle, therapists can better perceive what tissue is being pierced. In addition, no sensitive structures, such as nerves or arteries, are closely located to the plantar fascia, which is also an advantage. The precision of interventions for achieving optimal accuracy and precision in plantar fascia procedures is clinically relevant; however, it entails increased economic and training costs due to the use of ultrasound-guided approaches. Nevertheless, it is worth noting the palpation-guided approach also demonstrated clinically acceptable outcomes, suggesting its relevance in the clinical setting where resource constraints or advanced training in ultrasound may limit accessibility.

The current study has some limitations that should be considered. First, we consistently opted to initiate with landmark-guided techniques to mitigate the potential presence of learning bias, as conducting ultrasound-guided techniques first could have influenced the learning curve. The importance of visual feedback from ultrasound in the learning process has been well-established [25]. This fixed order (landmark-guided followed by ultrasound-guided) may induce bias due to fatigue in the later tasks. Second, our study was conducted on human anatomical samples. Due to their composition or structure, human cadavers are generally limited in terms of clinical reality. Furthermore, as the study was conducted on cadavers, the assessment of vascular structures was not feasible due to the absence of a pulse. Third, it is possible that performing the palpation-guided procedure before the ultrasound-guided procedure would have increased the skills of the therapists. Future studies considering these limitations should confirm or refute these results.

## 5. Conclusions

Ultrasound-guided techniques showed improved placement of the needle but not greater safety than landmark-guided techniques for targeting the interphase between the plantar fascia and the flexor digitorum brevis muscle. Thus, the ultrasound-guided procedure required more time than the landmark-guided procedure. In addition, the use of a handpiece accessory device to perform both ultrasound-guided and palpation-guided procedures required more time and a greater number of attempts, as compared to performing the needle approach without it. Future studies are needed to confirm these results in an in vivo sample and analyze their clinical implications.

## Figures and Tables

**Table 1:** Clinical characteristics (mean ± standard deviation) of the therapists and overall data on interventions.

	Mean (SD)
Experience with invasive techniques (years)	12.5 ± 4.3
Experience with ultrasound (years)	8.5 ± 2.3
Total needle procedures (n)	100
Palpation-guided/Ultrasound-guided (n)	50/50
With/without handpiece (n)	50/50
Distance to the target (mm)	1.83 (2.3)
Target contact (yes/no)	86/14
Time required (seconds)	36.5 (22.4)
Passes (total number)	2.3 (1.3)
Needle length outside (mm)	14.7 (3.3)

Abbreviations: n: number; mm: millimeters.

**Table 2:** Comparison of the measurements (mean ± standard deviation) between palpation-guided (n = 50) and ultrasound-guided (n = 50) procedures.

	Landmark-Guided	Ultrasound-Guided	Mean Difference (95% CI)	*p*	ES
Distance to interface between the plantar fascia and flexor digitorum brevis (mm) *	3.5 ± 2.2	0.2 ± 0.7	3.3 (2.7; 3.9)	<0.001	2.02
Longitudinal contact of the needle with interface (mm) *	0.6 ± 1.8	5.3 ± 2.2	−4.6 (−5.5; −3.8)	<0.001	2.34
Time required (seconds) *	19.1 ± 6.5	53.8 ± 18.9	34.8 (−40.5; −29.2)	<0.001	2.46
Passes (total number) *	1.7 ± 0.9	2.8 ± 1.5	−1.1 (−1.6; −0.6)	<0.001	0.89
Unwanted structures during needling					
None (reaching the interface)	41 (82%)	45 (90%)		0.249	0.12
Plantar fascia	9 (18%)	5 (10%)	-		
Flexor digitorum brevis	0 (0%)	0 (0%)			
Needle length outside (mm) *	13.1 ± 3.1	16.3 ± 2.7	−3.2 (−4.3; −2.0)	<0.001	1.01

Abbreviations: mm: millimetres; ES: effect size; CI: confidence interval. *: This variable was statistically significant.

**Table 3:** Comparison of the measurements (mean ± standard deviation) between landmark-guided procedures with (n = 25) and without (n = 25) the handpiece.

	With the Handpiece	Without the Handpiece	Mean Difference (95% CI)	*p*	ES
Distance to interface between the plantar fascia and flexor digitorum brevis (mm)	4.1 ± 1.7	2.9 ± 2.4	1.1 (−0.1; 2.3)	0.061	0.58
Longitudinal contact of the needle with the interface (mm)	0.3 ± 1.2	1.0 ± 2.2	−0.6 (−1.7; 0.4)	0.336	0.37
Time required (seconds) *	21.0 ± 3.7	17.1 ± 8.0	4.0 (0.4; 7.5)	0.005	0.63
Passes (total number)	1.7 ± 0.8	1.6 ± 0.9	0.1 (−0.4; 0.6)	0.450	0.12
Unwanted structures during needling					
None (reaching the interface)	22 (88%)	19 (76%)		0.269	0.16
Plantar fascia	3 (12%)	6 (24%)	-		
Flexor digitorum brevis	0 (0%)	0 (0%)			
Needle length outside (mm) *	14.6 ± 2.6	11.7 ± 2.9	2.9 (1.4; 4.5)	0.001	0.48

Abbreviations: mm: millimetres; ES: effect size; CI: confidence interval. *: This variable was statistically significant.

**Table 4:** Comparison of the measurements (mean ± standard deviation) between ultrasound-guided procedures with (n = 25) and without (n = 25) the handpiece.

	With the Handpiece	Without the Handpiece	Mean Difference (95% CI)	*p*	ES
Distance to interface between the plantar fascia and flexor digitorum brevis (mm)	0.4 ± 1.0	0.0 ± 0.0	0.4 (−0.6; 0.8)	0.077	0.57
Longitudinal contact of the needle with the interface (mm)	5.0 ± 2.6	5.5 ± 1.9	−0.5 (−1.8; 0.8)	0.900	0.22
Time required (seconds) *	59.6 ± 18.5	48.1 ± 17.8	11.6 (1.2; 21.9)	0.029	0.63
Passes (total number)	2.8 ± 1.6	2.8 ± 1.5	0.1 (−0.8; 0.9)	0.826	0.00
Unwanted structures during needling					
None (reaching the interface)	22 (88%)	23 (92%)		0.637	0.07
Plantar fascia	3 (12%)	2 (8%)	-		
Flexor digitorum brevis	0 (0%)	0 (0%)			
Needle length outside (mm)	16.0 ± 2.3	16.6 ± 3.0	−0.5 (−2.1; 1.0)	0.251	0.22

Abbreviations: mm: millimetres; ES: effect size; CI: confidence interval. *: This variable was statistically significant.

## Data Availability

The data presented in this study are available from the corresponding author on request.

## References

[1] Saggini R., Migliorini M., Carmignano S.M., Ancona E., Russo C., Bellomo R.G. (2018). Inferior Heel Pain in Soccer Players: A Retrospective Study with a Proposal for Guidelines of Treatment. BMJ Open Sport Exerc. Med..

[2] Riel H., Lindstrøm C.F., Rathleff M.S., Jensen M.B., Olesen J.L. (2019). Prevalence and Incidence Rate of Lower-Extremity Tendinopathies in a Danish General Practice: A Registry-Based Study. BMC Musculoskelet. Disord..

[3] Koc T.A., Bise C.G., Neville C., Carreira D., Martin R.L., McDonough C.M. (2023). Heel Pain—Plantar Fasciitis: Revision 2023. J. Orthop. Sports Phys. Ther..

[4] Schneider H.P., Baca J.M., Carpenter B.B., Dayton P.D., Fleischer A.E., Sachs B.D. (2018). American College of Foot and Ankle Surgeons Clinical Consensus Statement: Diagnosis and Treatment of Adult Acquired Infracalcaneal Heel Pain. J. Foot Ankle Surg..

[5] Babatunde O.O., Legha A., Littlewood C., Chesterton L.S., Thomas M.J., Menz H.B., van der Windt D., Roddy E. (2019). Comparative Effectiveness of Treatment Options for Plantar Heel Pain: A Systematic Review with Network Meta-Analysis. Br. J. Sports Med..

[6] Guimarães J.d.S., Arcanjo F.L., Leporace G., Metsavaht L.F., Conceição C.S., Moreno M.V.M.G., Vieira T.E.M., Moraes C.C., Gomes Neto M. (2023). Effects of Therapeutic Interventions on Pain Due to Plantar Fasciitis: A Systematic Review and Meta-Analysis. Clin. Rehabil..

[7] Grady J.F., Nagesh D., Smolinski T., Ostermann H.C. (2023). Plantar Fasciitis or Flexor Digitorum Brevis Myositis. J. Am. Podiatr. Med. Assoc..

[8] Draghi F., Ferrozzi G., Urciuoli L., Bortolotto C., Bianchi S. (2016). Hoffa’s Fat Pad Abnormalities, Knee Pain and Magnetic Resonance Imaging in Daily Practice. Insights Into Imaging.

[9] Sanchez-Ibáñez J.M. (2009). Clinical Course in the Treatment of Chronic Patellar Tendinopathy through Ultrasound Guided Percutaneous Electrolysis Intratissue (EPI^®^): Study of a Population Series of Cases in Sport. Ph.D. Thesis.

[10] Borrella-Andrés S., Malo-Urriés M., Pérez-Bellmunt A., Arias-Buría J.L., Rodríguez-Sanz J., Albarova-Corral M.I., González-Rueda V., Gallego-Sendarrubias G.M., Fernández-de-las-Peñas C., López-de-Celis C. (2022). Application of Percutaneous Needle Electrolysis Does Not Elicit Temperature Changes: An In Vitro Cadaveric Study. Int. J. Environ. Res. Public Health.

[11] Margalef R., Bosque M., Minaya-Muñoz F., Valera-Garrido F., Santafe M.M. (2021). Safety Analysis of Percutaneous Needle Electrolysis: A Study of Needle Composition, Morphology, and Electrical Resistance. Acupunct. Med..

[12] Gómez-Chiguano G.F., Navarro-Santana M.J., Cleland J.A., Arias-Buría J.L., Fernández-de-las-Peñas C., Ortega-Santiago R., Plaza-Manzano G. (2021). Effectiveness of Ultrasound-Guided Percutaneous Electrolysis for Musculoskeletal Pain: A Systematic Review and Meta-Analysis. Pain Med..

[13] Arias-Buría J.L., Borrella-Andrés S., Rodríguez-Sanz J., López-de-Celis C., Malo-Urriés M., Fernández-de-las-Peñas C., Gallego-Sendarrubias G.M., González-Rueda V., Pérez-Bellmunt A., Albarova-Corral I. (2023). Precision and Safety of Ultrasound-Guided versus Palpation-Guided Needle Placement on the Patellar Tendon: A Cadaveric Study. Life.

[14] Margalef R., Valera-Garrido F., Minaya-Muñoz F., Bosque M., Ortiz N., Santafe M.M. (2020). Percutaneous Needle Electrolysis Reverses Neurographic Signs of Nerve Entrapment by Induced Fibrosis in Mice. Evid.-Based Complement. Altern. Med..

[15] Mattiussi G. (2016). Treatment of Proximal Hamstring Tendinopathyrelated Sciatic Nerve Entrapment: Presentation of an Ultrasound-Guided “Intratissue Percutaneous Electrolysis” Application. Muscles Ligaments Tendons J..

[16] Neal J.M., Brull R., Chan V.W.S., Grant S.A., Horn J.-L., Liu S.S., McCartney C.J.L., Narouze S.N., Perlas A., Salinas F.V. (2010). The ASRA Evidence-Based Medicine Assessment of Ultrasound-Guided Regional Anesthesia and Pain Medicine. Reg. Anesth. Pain Med..

[17] Johnson A.N., Peiffer J.S., Halmann N., Delaney L., Owen C.A., Hersh J. (2017). Ultrasound-Guided Needle Technique Accuracy. Reg. Anesth. Pain Med..

[18] Gelfand H.J., Ouanes J.-P.P., Lesley M.R., Ko P.S., Murphy J.D., Sumida S.M., Isaac G.R., Kumar K., Wu C.L. (2011). Analgesic Efficacy of Ultrasound-Guided Regional Anesthesia: A Meta-Analysis. J. Clin. Anesth..

[19] Abrahams M.S., Aziz M.F., Fu R.F., Horn J.-L. (2009). Ultrasound Guidance Compared with Electrical Neurostimulation for Peripheral Nerve Block: A Systematic Review and Meta-Analysis of Randomized Controlled Trials. Br. J. Anaesth..

[20] Barrington M.J., Wong D.M., Slater B., Ivanusic J.J., Ovens M. (2012). Ultrasound-Guided Regional Anesthesia. Reg. Anesth. Pain Med..

[21] McVicar J., Niazi A.U., Murgatroyd H., Chin K.J., Chan V.W. (2015). Novice Performance of Ultrasound-Guided Needling Skills. Reg. Anesth. Pain Med..

[22] Boctor E.M., Choti M.A., Burdette E.C., Webster R.J. (2008). Three-dimensional Ultrasound-guided Robotic Needle Placement: An Experimental Evaluation. Int. J. Med. Robot. Comput. Assist. Surg..

[23] Stolka P.J., Foroughi P., Rendina M., Weiss C.R., Hager G.D., Boctor E.M. (2014). Needle Guidance Using Handheld Stereo Vision and Projection for Ultrasound-Based Interventions. Medical Image Computing and Computer-Assisted Intervention—MICCAI 2014, Proceedings of the 17th International Conference on Medical Image Computing and Computer-Assisted Intervention, Boston, MA, USA, 14–18 September 2014.

[24] López-de-Celis C., Fernández-de-Las-Peñas C., Malo-Urriés M., Albarova-Corral I., Arias-Buría J.L., Pérez-Bellmunt A., Rodríguez-Sanz J., González-Rueda V., Borella-Andrés S. (2023). Precision of Ultrasound-Guided versus Anatomical Palpation-Guided Needle Placement of the Ulnar Nerve at the Cubital Tunnel: A Cadaveric Study. Healthcare.

[25] Backhaus T., von Cranach M., Brich J. (2018). Ultrasound-Guided Lumbar Puncture with a Needle-Guidance System: A Prospective and Controlled Study to Evaluate the Learnability and Feasibility of a Newly Developed Approach. PLoS ONE.

